# Chemosensitivity, Cardiovascular Risk, and the Ventilatory Response to Exercise in COPD

**DOI:** 10.1371/journal.pone.0158341

**Published:** 2016-06-29

**Authors:** Michael K. Stickland, Desi P. Fuhr, Heather Edgell, Brad W. Byers, Mohit Bhutani, Eric Y. L. Wong, Craig D. Steinback

**Affiliations:** 1 Pulmonary Division, Department of Medicine, University of Alberta, Edmonton, Alberta, Canada; 2 G.F. MacDonald Centre for Lung Health, Covenant Health, Edmonton, Alberta, Canada; 3 Faculty of Physical Education and Recreation, University of Alberta, Edmonton, Alberta, Canada; Fondazione G. Monasterio, ITALY

## Abstract

COPD is associated with elevated cardiovascular risk and a potentiated ventilatory response to exercise. Enhanced carotid chemoreceptor (CC) activity/sensitivity is present in other clinical conditions, has been shown to contribute to sympathetic vasoconstrictor outflow, and is predictive of mortality. CC activity/sensitivity, and the resulting functional significance, has not been well examined in COPD. We hypothesized that CC activity/sensitivity would be elevated in COPD, and related to increased pulse wave velocity (a marker of CV risk) and the ventilatory response to exercise. Methods: 30 COPD patients and 10 healthy age-matched controls were examined. Participants performed baseline cardiopulmonary exercise and pulmonary function testing. CC activity was later evaluated by the drop in ventilation with breathing 100% O_2_, and CC sensitivity was then assessed by the ventilatory response to hypoxia (ΔVE/ΔSpO_2_). Peripheral arterial stiffness was subsequently evaluated by measurement of pulse wave velocity (PWV) using applanation tonometry while the subjects were breathing room air, and then following chemoreceptor inhibition by breathing 100% O_2_ for 2 minutes. Results: CC activity, CC sensitivity, PWV and the ventilatory response to exercise were all increased in COPD relative to controls. CC sensitivity was related to PWV; however, neither CC activity nor CC sensitivity was related to the ventilatory response to exercise in COPD. CC inhibition by breathing 100% O_2_ normalized PWV in COPD, while no effect was observed in controls. Conclusion: CC activity and sensitivity are elevated in COPD, and appear related to cardiovascular risk; however, CC activity/sensitivity does not contribute to the potentiated ventilatory response to exercise.

## Introduction

Chronic Obstructive Pulmonary Disease (COPD) is characterized by progressive, partially reversible airway obstruction, marked exertional dyspnea and exercise intolerance[1]. While COPD was originally considered to be a disease specific to the lung, it is now appreciated that there are substantial systemic manifestations, such as cardiovascular disease, that develop as a consequence of COPD[1, 2]. COPD is associated with elevated risk for cardiovascular (CV) morbidity and mortality[3, 4]. Furthermore, COPD patients are almost three times more likely to die of heart failure than smokers not diagnosed with COPD[5] indicating that the CV pathophysiology of COPD is not simply related to smoking history.

COPD patients have important modifications to autonomic nervous system regulation that would contribute to CV disease. In particular, both hypoxemic and non-hypoxemic COPD patients have evidence of increased sympathetic nerve activity[6–12]. Clinical relevance regarding the negative effects of chronic sympathetic activation in COPD has recently been demonstrated, as the degree of resting sympathetic activity is predictive of mortality and hospitalizations in COPD[12]. The mechanism(s) causing elevated sympathetic activity, and subsequent CV disease in COPD have been poorly characterized; however, greater carotid chemoreceptor (CC) activity/sensitivity may play a role. The CC is generally considered to be the major oxygen sensor in the body; however, the CC is sensitive to a variety of stimuli, including inflammation (IL-6[13], TNF-α[13]), angiotensin II[14] and increased reactive oxygen species, and COPD is associated with increases in both inflammation and reactive oxygen species[15, 16]. CC activation/stimulation elicits significant increases in sympathetic outflow[17, 18], which can have important negative CV consequences including tonic vasoconstriction[19, 20]. There is some evidence that the CC is also sensitized in COPD. COPD patients have been found to have an exaggerated heart rate response to hypoxia[21], while some[22], but not all studies[21] have found evidence of greater ventilatory drive in response to hypoxia; however, CC activity/sensitivity has not be well studied in COPD.

The CC has also been shown to contribute to the regulation of ventilation during exercise[23, 24]. COPD patients are characterized as having potentiated dyspnea, as well as an exaggerated ventilatory response to exercise regardless of COPD severity. The finding that even mild COPD patients show a potentiated ventilatory response to exercise would suggest that this exaggerated ventilatory response may not be fully explained by lung function/mechanics, and that other determinants such as enhanced CC activity/sensitivity may play a role.

Importantly, CV comorbidities such as hypertension, heart failure, as well as sleep apnea can potentiate CC activity/sensitivity, whereas standard medication for management for CV disease such as β-blockers and angiotensin receptor blockers [25] can supress the CC and/or ventilation [26–28]. Thus, in order to properly determine if CC activity/sensitivity is elevated in COPD, and the possible CV consequences, a sample of patients who have been carefully screened for CV comorbidities and CV medications is required. Thus, the purpose of the present study was to examine CC activity/sensitivity in a group of non-hypoxic COPD patients, and age-matched controls, who were carefully screened for CV comorbidities, sleep apnea, and CV medications. We hypothesized that CC activity/sensitivity would be elevated in COPD. As enhanced CC activity/sensitivity has also been shown to contribute to greater sympathetic vasoconstrictor outflow, we hypothesized that CC activity/sensitivity would be related to arterial stiffness as evaluated by pulse wave velocity, and that transient CC inhibition with short-term inspired O_2_ would reduce arterial stiffness. In addition, as the CC has been shown to play a role in ventilation during exercise, we hypothesized that the enhanced CC activity/sensitivity would be related to the potentiated ventilatory response to exercise observed in COPD.

## Methods

The present study was approved by the University of Alberta Health Research Ethics Board (Biomedical Panel). Written informed consent was obtained prior to any research procedures. This paper was part of a larger project examining cardiovascular risk in COPD, and a publication examining arterial stiffness and physical activity has been previously published [29]. Control subjects (n = 10) and some of the COPD patients (n = 20) previously examined were also included in the current paper.

### Subjects

Thirty non-hypoxic COPD patients and 10 age-matched controls were recruited. COPD patients were selected using the American Thoracic Society criteria of irreversible post-bronchodilator airflow obstruction (i.e. FEV_1_/FVC below the lower limit of normal predicted from height, age, sex)[30] and a smoking history >10 pack-years (see Table 1). Medical history of each participant was carefully reviewed by a study physician to exclude supplemental oxygen therapy, diabetes, cardiovascular disease, vasoactive medications, body mass index (BMI) ≥32, severe inflammatory disorders (such as connective tissue disease), and recent participation in pulmonary rehabilitation. The lack of CV disease was also confirmed by a normal resting blood pressure and 12-lead ECG, as well as normal blood pressure and 12-lead ECG responses to maximal exercise. In addition, no COPD patient had a COPD exacerbation within the previous 6 months. Age-matched controls met the same criteria, yet had normal lung function and <10 pack year smoking history. All subjects were screened for sleep apnea, as evaluated by an overnight home sleep monitor (ApneaLink Plus, ResMed Ltd., Bella Vista, Australia), and no subject showed evidence of significant sleep apnea (Respiratory Disturbance Index <15).

**Table 1:** Subject characteristics (mean ± SD).

	Age-Matched Controls	COPD Patients
Subjects	10	30
Age (yrs)	67.5 ± 5.1	64.9 ± 7.9
Height (cm)	170 ± 11	169 ± 11
BMI (kg/m^2^)	26.8 ± 2.6	26.5 ± 4.8
Smoking history (pack years)	3.4 ± 4.7	39.6 ± 17.8*
FEV_1_ (L)	2.8 ± 0.7	1.7 ± 0.7*
FEV_1_ (% pred)	102.8 ± 13.8	57.1 ± 23.0*
FVC (% pred)	105.3 ± 15.3	95.9 ± 22.8
FEV_1_/FVC ratio	75.1 ± 5.2	46.8 ± 14.0*
TLC (% pred)	N/A	116.3 ± 22.3
FRC (% pred)	N/A	133.0 ± 42.3
RV (% pred)	N/A	141.2 ± 55.3
DLCO (% pred)	N/A	66.2 ± 18.3
MRC Dyspnea Scale (1–5)	1.0 ± 0.0	2.1 ± 0.9*

Note: Values are means ± SD

* = p <0.05 vs. control.

Definition of abbreviations: BMI, body mass index; FEV_1_, forced expired volume in 1 second; FVC, forced vital capacity; FEV_1_/FVC ratio, forced expiratory volume in one second to forced vital capacity ratio; TLC, total lung capacity; FRC, functional residual capacity; DLCO, diffusion capacity for carbon monoxide; MRC, Medical Research Council; %pred, percent predicted.

### Study Protocol

All data for each participant were obtained over two testing days within a 30 day period. For each day, participants ate a light breakfast, and reported to the laboratory between 7:00 and 10:00 AM. Participants abstained from caffeine, exercise, alcohol and respiratory medications for at least 6 hrs prior to testing[31].

On the first testing day, participants completed a brief medical history and the Medical Research Council (MRC) dyspnea scale[32]. All participants performed standardized spirometry[30], while COPD patients completed a full pulmonary function test. Each participant subsequently performed a cardiopulmonary exercise test (CPET).

### Cardiopulmonary Exercise Test (CPET)

The CPET was performed on a treadmill as previously described[33]. Cardiorespiratory data were collected using a Vmax metabolic testing system (Vmax Spectra V29 System; SensorMedics, Yorba Linda, CA), and data are reported in Table 2. Peak oxygen consumption (VO_2peak_) and peak heart rate were defined as the highest values obtained during a 20 s period during the test. Inspiratory capacity maneuvers were conducted at rest and every two minutes up to peak exercise. Breathing reserve was calculated as: (predicted maximum voluntary ventilation (FEV_1_ x 35)—peak minute ventilation) / predicted maximum voluntary ventilation x 100.

Subjects returned to the lab on a second day for an assessment of vascular and chemoreceptor activity/sensitivity. Throughout the procedures, subjects lay supine and breathed through a mouthpiece with the nose occluded. Inspired gas was humidified (HC 150; Fisher and Paykel Healthcare, Auckland, New Zealand) and delivered continuously using a flow-through system to prevent rebreathing of expired gas (flow: 0.5–1.0 l/s). Ventilation was measured by a pneumotachometer (3700 series; Hans Rudolph, Kansas City, MO) just distal to the mouthpiece. Expired CO_2_ and O_2_ (mmHg) were measured (Analyzers 17630/17625; Vacumed, Ventura, CA) continuously from a small sample port off of the mouthpiece. Arterial oxygen saturation (SpO_2_) was estimated with pulse oximetry (N-595; Nellcor Oximax, Boulder, CO) using a forehead sensor. Heart rate was recorded with a single-lead ECG (lead II, Dual Bio Amp; ADInstruments), and blood pressure was monitored using finger photoplethysmography (Finometer model 2; Finapres, Amsterdam, The Netherlands). Data were recorded and integrated through a data acquisition system and sampled at a rate of 1 k·s^-1^ (Powerlab 16/30, ADInstruments, New South Wales, Australia) and analyzed offline using the associated software (LabChart 7.0, ADInstruments, New South Wales, Australia).

**Table 2:** Cardiopulmonary exercise data (mean ± SD).

	Age-Matched Controls	COPDPatients
Subjects	10	30
Peak VO_2_ (ml·kg^-1^·min^-1^)	30.1 ± 8.7	19.4 ± 3.5*
Peak VO_2_ (L·min^-1^)	2.2 ± 0.6	1.4 ± 0.5*
Peak VCO_2_ (L·min^-1^)	2.1 ± 0.9	1.5 ± 0.5*
Peak RER	1.1 ± 0.1	1.1 ±0.1
Peak V_E_ (L·min^-1^)	80.2 ± 39.2	51.7 ± 15.3*
Peak PETCO_2_	36.6 ± 4.6	34.2 ± 4.2
Peak PETO_2_	96.6 ± 6.4	99.4 ± 6.4
Peak SpO_2_ (%)	92.7 ± 2.0	91.1 ± 4.3
Peak HR (beats·min^-1^)	161.5 ± 13.8	127.1 ± 16.7*
Mean V_E_/VO_2_ Slope	24.0 ± 6.6	25.5 ± 5.7
Mean V_E_/VCO_2_ Slope	27.7 ± 1.0	32.1 ± 6.5*
Breathing Reserve (%)	29.3 ± 33.4	1.6 ± 31.1*
Change in inspiratory capacity (baseline to peak exercise, L)	-0.15 ± 0.37	-0.36 ± 0.35*

Note: Values are means ± SD

* = p <0.05 vs. control.

Definition of abbreviations: VO_2_, oxygen uptake; VCO_2_, carbon dioxide production; RER, respiratory exchange ratio; V_E_, ventilation; PETCO_2_, end tidal carbon dioxide; PETO_2_, end tidal oxygen; SpO_2_, oxygen saturation measured by pulse oximeter; HR, heart rate.

### Arterial Stiffness

Following instrumentation, the subject lay supine on a bed and rested for at least 10 minutes. Once blood pressure and heart rate were stable, resting carotid-radial pulse wave data were collected. Pulse waves were gathered simultaneous using applanation tonometry (Mikro-tip Catheter Transducers model SPT-301, Millar Instruments, Inc., Houston, Texas) from the carotid and radial arteries. PWV was calculated as *PWV = D∙Δt*^-*1*^, where *D* was the distance (m) between sites and *Δt* was the time difference (s) between pulse waves using the foot-to-foot method[31]. Distance was measured on the surface of the body using a tape measure beginning at the sternal notch and extending to the recording sites of the carotid and radial arteries[31]. Mean PWV was calculated as the average of at least 10 consecutive beats in order to cover a full respiratory cycle[31].

### CC Activity

Basal CC activity was then evaluated by examining the transient decrease in resting ventilation in response to hyperoxia[23, 34]. After 5 minutes of resting baseline measurements breathing normoxic gas, subjects breathed hyperoxia (F_I_O_2_ = 1.0) for 2 minutes through our non-re-breathing system. The difference between the baseline ventilation and the nadir (i.e. lowest 15 second bin) of any ensuing decrease in ventilation was taken as the index of the basal CC activity[35, 36]. PWV and other cardiorespiratory data were also collected in the last 30 seconds of the first minute of breathing hyperoxia, as this time-frame has been shown to correspond to reductions in muscle sympathetic nerve activity secondarily to CC inhibition from hyperoxia[37]. Of note, prolonged exposure to hyperoxia was avoided as long-term hyperoxia can act as a central stimulant[38] and cause other secondary effects.

### CC Sensitivity

Hypoxia is a potent CC stimulant and used to evaluate CC sensitivity[39, 40]. After 10 minutes of recovery and 5 minutes of resting baseline measurements breathing room air, N_2_ was blended into the inspired air to cause step drops in arterial saturation to 90% and 85% for 3 minutes each. Carotid chemosensitivity was then evaluated by graphing the slope of the increase in ventilation (ΔVE) relative to the drop in arterial saturation (ΔSpO_2_). Arterial saturation was targeted as opposed to end-tidal O_2_ because of the uncertainty regarding the accuracy of estimating arterial blood gas data from end-tidal in patients with lung disease.

### Statistical Analysis

For all inferential analyses, the probability of type I error was set at 0.05. Between group comparisons were made using unpaired t-tests. To evaluate changes in cardiorespiratory variables with hyperoxia, a repeated measured ANOVA was conducted (SPSS version 21, IBM, Armonk, NY). Within the COPD patients, pearson-product-moment-correlations were calculated to describe the strength of relationships between CC activity/sensitivity and other cardiorespiratory variables.

## Results

There was no between-group difference in age, height or BMI between COPD and controls (see Table 1). As expected, COPD patients had airflow obstruction and greater resting dyspnea.

COPD patients had lower VO_2peak_, reduced breathing reserve, and evidence of dynamic hyperinflation as demonstrated by a reduction in inspiratory capacity with incremental exercise (see Table 2).

### Chemoreceptor activity

**Fig 1:**
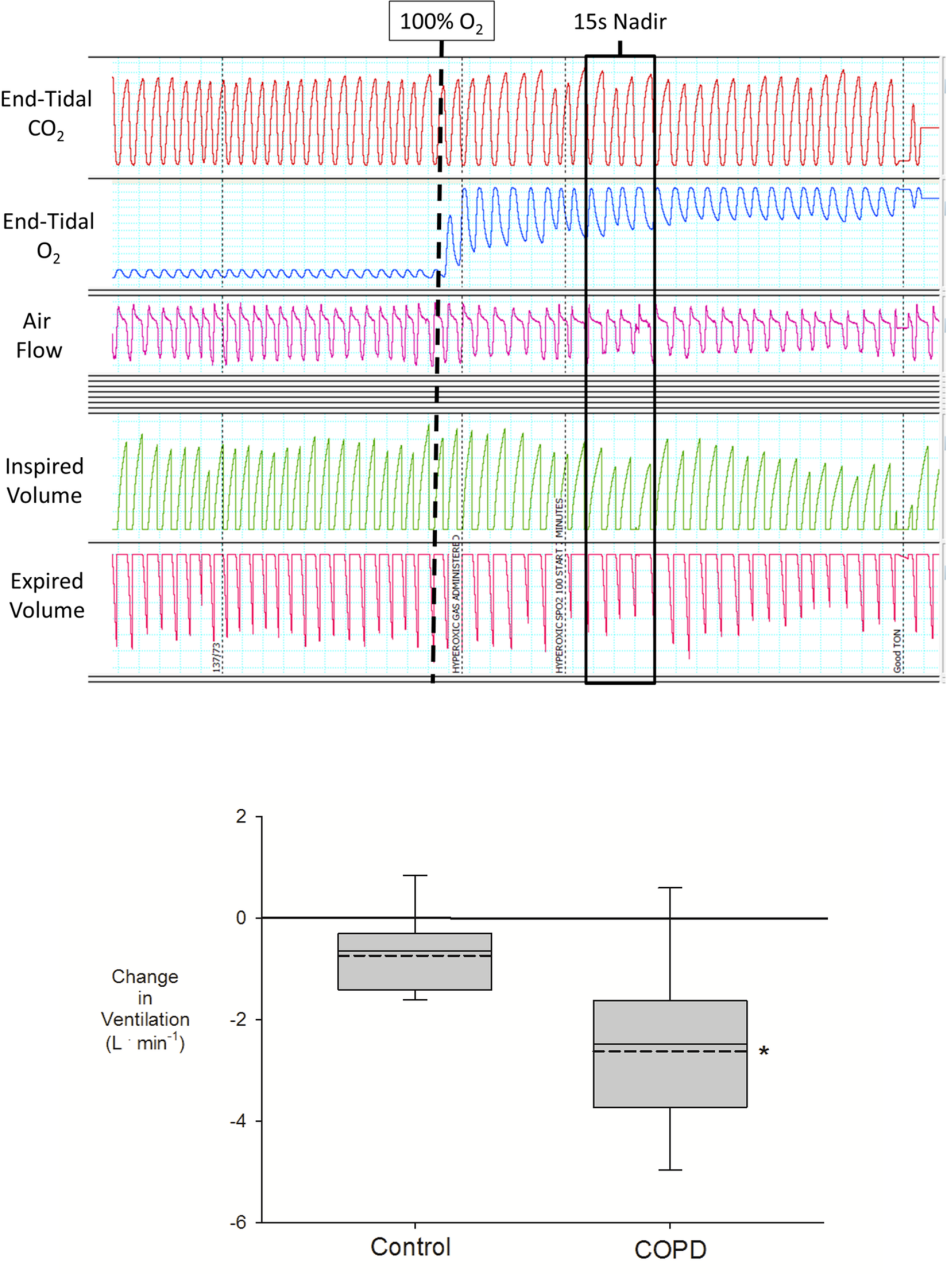
Representative trace of the transient reduction in minute ventilation in response to breathing 100% oxygen (top). Mean response to the transient reduction in minute ventilation in response to breathing 100% oxygen in Controls and COPD (Bottom) NOTE: dashed lines correspond to the mean response, * = p<0.05 vs. control.

Resting minute ventilation was higher in COPD patients as compared to controls (9.0 ± 2.4 vs. 7.2 ± 1.8 L/min, p = 0.035). The transient reduction in minute ventilation while breathing 100% O_2_ was greater in COPD patients as compared to controls, indicating greater tonic chemoreceptor activity in COPD (see Fig 1).

### Chemoreceptor sensitivity

**Fig 2:**
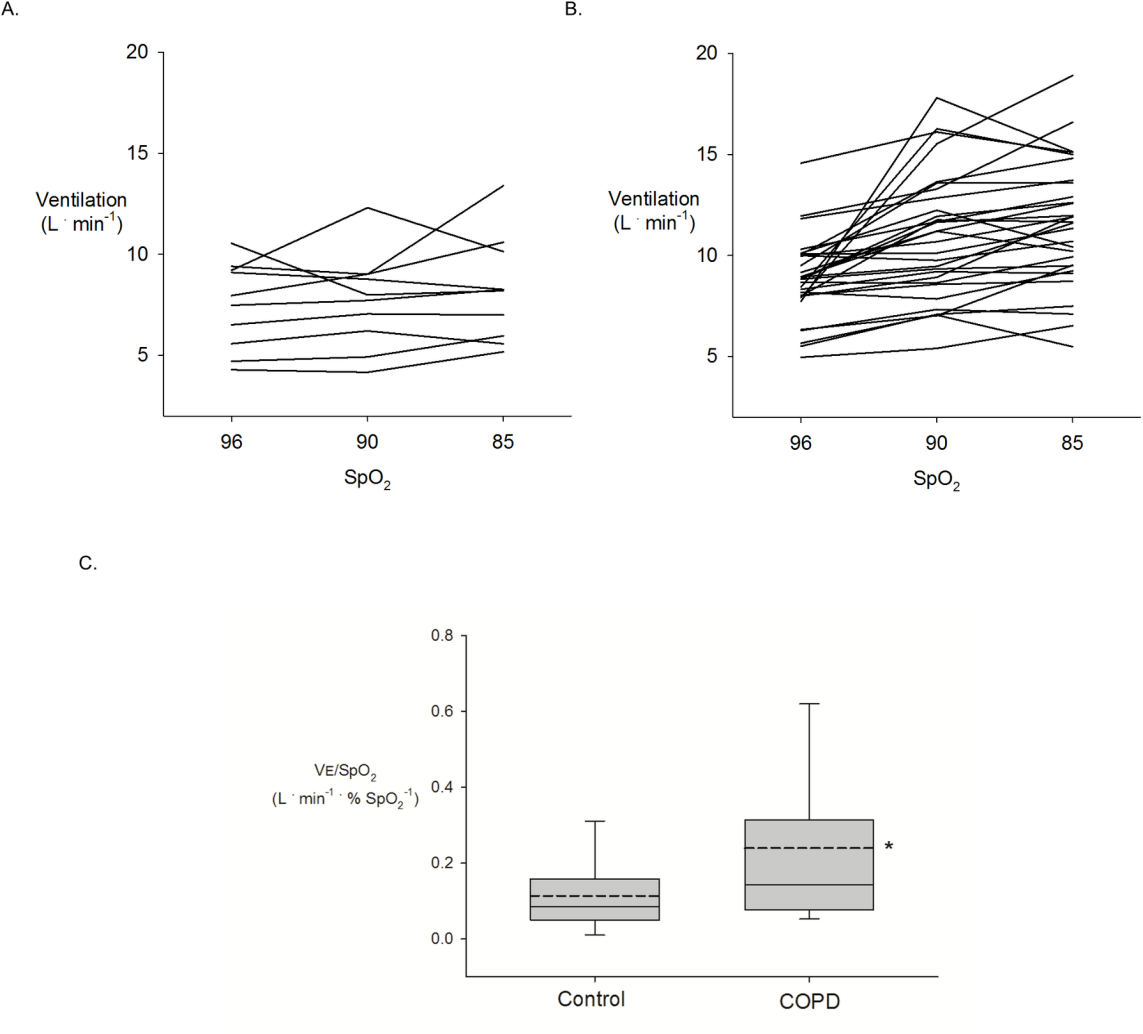
Individual ventilatory response to the reduction in arterial saturation in controls (A) and COPD (B), and the mean grouped ventilatory response (ΔVE / ΔSpO_2_ slope) in controls and COPD (C). NOTE: dashed lines correspond to the mean response, * = p<0.05 vs. control.

The slope of the ventilatory response to hypoxia (i.e. ΔVE/ΔSpO_2_) in COPD and controls are shown in Fig 2. Mean ΔVE/ΔSpO_2_ was greater in COPD as compared to controls, indicating greater chemosensitivity in COPD (see Fig 2).

### Chemoreceptor activity/sensitivity, pulse wave velocity, and exercise responses

**Fig 3:**
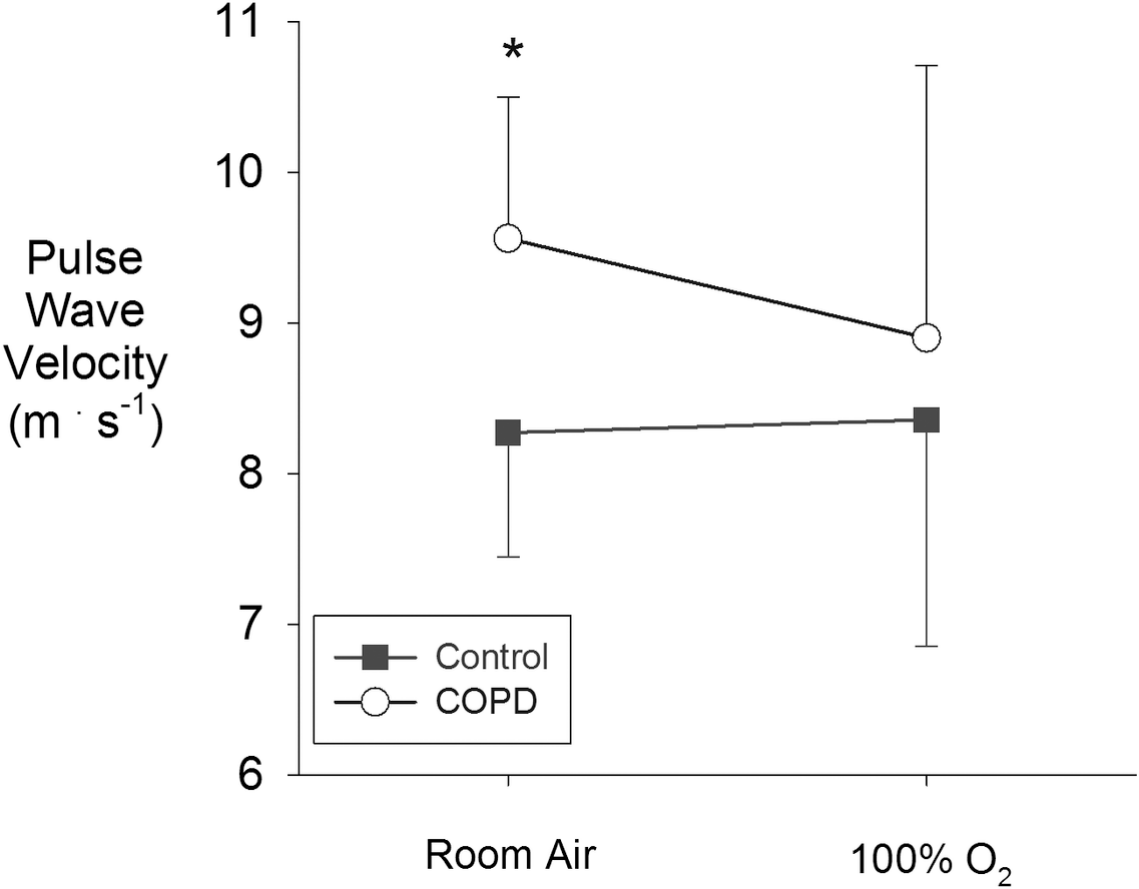
Pulse wave velocity at rest, and during 2 minutes of breathing 100% O_2_ in controls and COPD patients. NOTE: p<0.05 vs. control.

Resting PWV data in COPD and controls as well as the response to breathing 100% O_2_ are shown in Fig 3. Resting PWV was higher in COPD (9.6±0.9 m/s) as compared to controls (8.3±0.8 m/s; p<0.05). Breathing 100% O_2_ for 2 minutes significantly reduced PWV in COPD patients (8.9±1.8 m/s; p<0.05) but did not reduce PWV in controls (8.4±1.4 m/s). Thus, the difference in PWV observed at rest between COPD and controls was normalized while breathing 100% O_2_. Blood pressure was unaffected by 100% O_2_ in both groups, while 100% O_2_ reduced heart rate in COPD but not controls (see Table 3).

**Table 3:** Cardiorespiratory responses to one minute of breathing hyperoxia (mean ± SD).

	Baseline		Hyperoxia	
	Controls	COPD	Control	COPD
PETCO_2_ (mmHg)	39.8 ± 4.6	37.0 ± 4.2	35.8 ± 7.5	37.7 ± 4.4
PETO_2_ (mmHg)	106.8 ± 10.4	107.7 ± 7.9	595.3 ± 15.6*	593.1 ± 11.1*
SpO_2_ (%)	96.3 ± 1.4	95.4 ± 2.1	99.8 ± 0.3*	99.6 ± 1.7*
Heart rate (bpm)	63.0 ± 7.8	70.2 ± 12.3	61.9 ± 8.5	65.8 ± 12.4*
Mean arterial pressure (mmHg)	84.2 ± 7.9	90.0 ± 8.9	83.6 ± 9.9	89.0 ± 8.7
Systolic blood pressure (mmHg)	117.0 ± 11.2	120.6 ± 12.9	119.6 ± 13.5	118.5 ± 12.0
Diastolic blood pressure (mmHg)	67.8 ± 7.9	74.7 ± 9.2	68.3 ± 9.2	74.2 ± 9.0

Note: Values are means ± SD

* = p <0.05 vs. normoxia.

Definition of abbreviations: PETCO_2_, end tidal carbon dioxide; PETO_2_, end tidal oxygen; SpO_2_, oxygen saturation measured by pulse oximeter.

**Fig 4:**
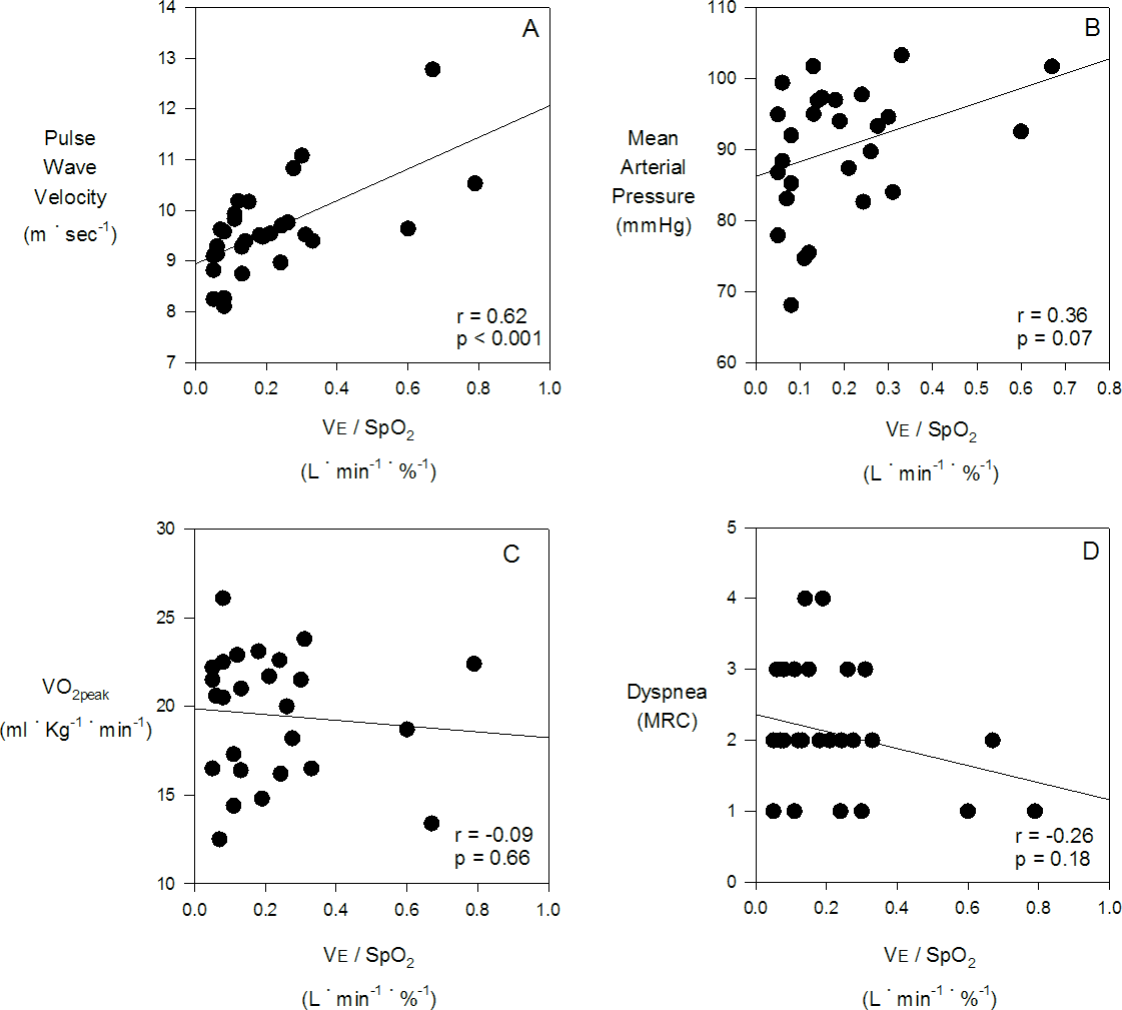
Regression analysis of the increase in ventilation in response to stepwise reductions in arterial saturation with hypoxia (ΔVE / ΔSPO_2_ slope) in COPD vs. pulse wave velocity (A), mean arterial pressure (B), VO_2peak_ (C), and Dyspnea (D).

Within the COPD patients, the ventilatory response to hypoxia (ΔVE / ΔSPO_2_ slope) was correlated with baseline PWV (r = 0.62, p <0.001, see Fig 4); however, the ventilatory response to hypoxia was not related to baseline MAP (r = 0.36, p = 0.07), VO_2peak_ (r = -0.09, p = 0.66), Dyspnea (r = -0.26, p = 0.18), nor FEV1 (r = 0.02, p = 0.91).

Within the COPD patients, the decrease in ventilation in response to hyperoxia (Delta VE) was not correlated with PWV (r = 0.04, p = 0.83), MAP (r = 0.03, p = 0.88), Dyspnea (r = -0.05, p = 0.80), VO_2peak_ (r = -0.24, p = 0.25), nor FEV_1_ (r = -0.11, p = 0.59).

### Chemoreceptor activity/sensitivity and ventilatory response to exercise

The VE/VCO_2_ response to exercise was unrelated to: the ventilatory response to hypoxia (r = 0.32, p = 0.12), the ventilatory response to hyperoxia (r = 0.10, p = 0.65), nor FEV_1_(r = -0.04, p = 0.85).

## Discussion

The data from the current study demonstrate elevated CC activity and sensitivity in COPD patients. Importantly, the elevated CC activity/sensitivity in COPD appears clinically important, as CC sensitivity was directly related to arterial stiffness in COPD patients, while CC inhibition with hyperoxia normalized the observed elevated arterial stiffness. These results suggest that increased carotid chemoreceptor activity and sensitivity may contribute to elevated cardiovascular risk in COPD.

Despite well-characterized elevations in CC activity/sensitivity in other clinical conditions, there are relatively few studies examining the CC in COPD. Further, the limited number of previous investigations focused on more severe COPD patients, who may have more complex responses to hypoxia. Erbland et al.[22] found that COPD patients (mean FEV_1_ = 0.7 ± 2L) had a greater ventilatory drive to hypoxia as compared to controls[22], whereas Miyamoto et al. found no difference in ventilatory response to hypoxia in COPD patients (mean FEV_1_ = 45 ± 21%) as compared to controls, but did find a potentiated heart rate response to hypoxia in COPD[21]. Bradley et al.[41] observed that both the occlusion pressure and ventilatory response to hypoxia was reduced in COPD patients who were chronically hypoxemic as opposed to those who were not[41], whereas Erbland et al.[22] did not observe a relationship between hypoxic ventilatory drive and chronic hypoxemia. Because of the underlying severity of the lung disease in the previous studies, some patients approached their maximum voluntary ventilation (as estimated by spirometry) during resting hypoxic/hypercapneic challenges[22], which prompted some patients to terminate the trials because of severe dyspnea. Importantly in the current study, patients were well below their estimated maximum voluntary ventilation as well as their peak minute ventilation achieved during the cardiopulmonary exercise test, indicating that a mechanical limitation to ventilation during the hypoxic challenge was unlikely.

We suggest that the approach taken in the current study provides important information regarding CC activity/sensitivity in COPD and the linkage to cardiovascular disease risk. Firstly, the present study carefully screened for CV co-morbidities such as sleep apnea, hypertension and previous CV disease, all of which are well-known to modify CC activity/sensitivity. Further, in contrast to previous work, patients were excluded if they were taking medications such as β-blockers and angiotensin receptor blockers, as these can suppress the CC and/or ventilation [26–28]. By rigorously excluding common conditions such as hypertension and sleep apnea, we were able to examine the direct effect of COPD on CC activity/sensitivity independent of associated co-morbidities.

The patients recruited in the current study were typically less severe than previous work[22, 24, 41]. As mentioned above, chronic hypoxemia may[41], or may not[22] blunt the ventilatory response to acute hypoxia, whereas hypercapnea likely potentiates the ventilatory response to hypoxia. In the current study, none of the patients had resting hypoxemia, nor hypercapnea, and most did not develop hypoxemia even at VO_2peak_. None-the-less, the current data clearly show CC hyperactivity and sensitization in COPD, with the latter associated with stiffer arteries in these patients. Thus, CC hyperactivity and sensitization may evolve early in the development of COPD and play a role in the progression of cardiovascular comorbidities.

Conditions such as chronic heart failure (CHF) and hypertension, common co-morbidities with COPD, have been shown to have both greater tonic CC activity[20, 42], and enhanced CC sensitivity[43–45]. Enhanced CC activity/sensitivity is clinically significant, as CHF patients with elevated chemosensitivity have greater mortality as compared to CHF patients with normal chemosensitivity[44]. It is likely that a CC-mediated elevation in sympathetic nerve activity explains the link between CC sensitivity and mortality in CHF[46]. Arterial stiffness is determined by material properties of the arterial wall (i.e. elastin/collagen) as well as vessel diameter (i.e. smooth muscle tone)[31], which is influenced by sympathetic nerve activity[47]. Indeed resting sympathetic nerve activity has been shown to be correlated with arterial stiffness[48] and arterial wall thickening[49], and it was for this reason that a possible link between CC activity/sensitivity and PWV was investigated. In the present study, COPD patients had elevated PWV as compared to controls, and CC sensitivity was found to be related to PWV in COPD patients (r^2^ = 0.38, p<0.001, see Fig 4). Importantly, CC sensitivity appears to be a stronger predictor of PWV than previously reported associations comparing arterial stiffness with FEV_1_ (r^2^ = 0.11)[50], IL-6 (r^2^ = 0.09)[50] and emphysema severity (r^2^ = 0.22)[51]. These results suggest that the increased CC sensitivity in COPD may be contributing to the increase in resting sympathetic activity previously reported in COPD[6–12], which in turn would increase PWV and CV risk.

Previous work has also shown that when the CC activity/sensitivity is elevated, CC inhibition results in vasodilation and an improvement in CV function[19, 20]. Similarly, if the CC is contributing to an elevation in sympathetic nervous activity in COPD, inhibition of the CC would be expected to reduce PWV. Consistent with this hypothesis, CC inhibition with short-term hyperoxia caused a reduction in PWV in COPD patients, while no effect was observed in healthy controls. This is the first demonstration that PWV can be acutely improved in COPD, and is consistent with previous work showing that breathing hyperoxia (F_I_O_2_ = 0.31) reduces mean arterial pressure and improves autonomic function as assessed by heart rate variability in severe COPD patients[52]. Breathing short-term 100% O_2_ would not affect arterial wall properties, indicating that vessel diameter must have been altered with 100% O_2_. These findings indicate that the increased CC activity in COPD is important as it contributes to the increased basal PWV in COPD. Follow-up studies are required to more carefully target the CC in COPD, and evaluate sympathetic nervous activity and cardiovascular function.

COPD patients typically demonstrate an exaggerated ventilatory response to exercise (as evaluated by an increased VE/VCO_2_ response)[53], which in addition to airflow obstruction, would potentiate dyspnea on exertion, and impair exercise tolerance. Studies have shown that the CC plays a role in exercise ventilation[23, 24], suggesting that increased CC activity/sensitivity may contribute to the potentiated ventilatory response in COPD. Contrary to our hypothesis, there was no association between CC activity/sensitivity and the ventilatory response to exercise in COPD, suggesting that the potentiated ventilatory response to exercise in COPD is unlikely to be explained by increased chemosensitivity.

### Considerations

Prolonged breathing of hyperoxia was avoided as it is acknowledged that long-term hyperoxia can cause secondary effects including increased oxidative stress, central stimulation and vasoconstriction[38, 54, 55]. This may explain why prolonged high O_2_ breathing has been shown to cause increased arterial stiffness, as evaluated by augmentation index, in severe COPD patients[56]. These findings indicate that caution should be taken when considering prolonged high O_2_ in COPD, as hyperoxic conditions may actually increase CV risk.

Participants in the present study were carefully selected to exclude co-morbidities that can affect CC activity/sensitivity. While this approach allowed us to examine the effect of COPD on CC activity/sensitivity, the narrow inclusion criteria limits the generalizability of the findings to the broader COPD population. Arterial blood gases were not collected in our COPD patients. However, SpO_2_ values were maintained up to peak exercise in all participants, and end-tidal CO_2_ values were normal at rest in the COPD patients, indicating that baseline hypoxemia and/or hypercapnea was unlikely.

Arterial stiffness was evaluated using carotid-radial PWV as opposed to carotid-femoral PWV.

Central (carotid-femoral) PWV is mainly affected by the intrinsic structural composition of the large arteries[57]. Carotid-radial PWV reflects peripheral arterial stiffness, and the resistive (i.e. peripheral) arteries are under more muscular/sympathetic control, and therefore would be more sensitive to acute modulations of sympathetic outflow as compared to central PWV. As central PWV is predictive of CV events/mortality[58, 59], follow-up studies examining carotid-femoral PWV would help evaluate the significance of the CC on CV risk.

In the current study, CC activity/sensitivity was evaluated by changes in ventilation. Importantly, FEV_1_ was not related to either the ventilatory response to hypoxia nor the drop in ventilation with hyperoxia within the COPD patients, suggesting that it is not the decrements in lung function per se that are driving the potentiated ventilatory response to hypoxia/hyperoxia in COPD.

The potential influence of the central chemoreceptors cannot be determined; however, it is unlikely that the present findings are explained by differences in central chemoreception. Carotid body denervation in humans abolishes the ventilatory response to hypoxia[60], indicating that the CC plays the key role in the hypoxic response. Likewise, it is unlikely that short-term central hyperoxia, per se, would result in a reduction in ventilation and/or PWV because: (a) substantial levels of hypoxemia are required to stimulate sympathetic outflow[61] or ventilation[62] via central mechanisms, and all our subjects were non-hypoxemic at rest; and (b) central hyperoxia in isolation increases, not decreases, ventilation[38].

This study examined CC activity/sensitivity in a group of COPD patients, and age-matched controls, who were carefully screened for CV comorbidities and sleep apnea. COPD patients were found to have enhanced CC sensitivity as demonstrated by a potentiated ventilatory response to hypoxia, as well as greater resting CC activity, as demonstrated by the reduction in ventilation following CC inhibition by breathing 100% O_2_. The enhanced CC activity/sensitivity in COPD appears clinically important, as CC sensitivity was related to baseline arterial stiffness in COPD, and CC inhibition with 100% O_2_ reduced arterial stiffness in COPD patients down to age-matched healthy control values. Combined, these results suggest that carotid chemoreceptor hyperactivity and sensitivity may contribute to increased cardiovascular risk in COPD.

## Supporting Information

S1 File This is the Chemo Paper Data De-identified June 2016_Final excel file containing all supporting data for the manuscript. (XLSX)
